# Most of the pelvic floor muscle functions in women differ in different body positions, yet others remain similar: systematic review with meta-analysis

**DOI:** 10.3389/fmed.2023.1252779

**Published:** 2023-11-06

**Authors:** Lu Huang, Zhi-Yuan Zhang, Hong Liu, Min Gao, Xiao-Qi Wang, Xiao-Qin Duan, Zhong-Liang Liu

**Affiliations:** ^1^School of Nursing, Jilin University, Changchun, China; ^2^Department of Rehabilitation, The Second Hospital of Jilin University, Changchun, China

**Keywords:** evaluation study, maximum voluntary contractions, pelvic floor, position, stress urinary incontinence

## Abstract

**Objectives:**

This systematic literature review and meta-analysis aimed to determine the effect of body position on the measurement of pelvic floor muscle (PFM) contractility and to analyze the influential factors.

**Data sources:**

Five databases (PubMed, Web of Science, EMBASE, Cochrane Library and Scopus) were searched for relevant studies published up to 12nd October 2023.

**Study selection or eligibility criteria:**

Included cross-sectional studies had to involve the assessment of pelvic floor muscle function in at least two positions.

**Study appraisal and synthesis methods:**

We calculated standardized mean difference (SMD) with 95% confidence intervals (CI) to ascertain the potential effect of body position on outcomes.

**Results:**

In total, we included 11 cross-sectional studies to ascertain the potential effect of body position on outcomes. There was no statistical difference in the results of maximum voluntary contraction (MVC) of the pelvic floor muscles when assessed in between supine and standing positions (SMD −0.22; 95% CI −0.72 to 0.28; *p* = 0.38). The results of the meta-analysis showed significantly larger values of resting voluntary contractions (RVC) measured in the standing position compared to the supine position (SMD −1.76; 95% CI −2.55 to −0.97; *p* < 0.001). Moreover, pelvic floor muscle movement during pelvic floor muscle contraction in the standing position was significantly better than that measured in the supine position (SMD −0.47; 95% CI −0.73 to 0.20; *P* < 0.001).

**Conclusion:**

The results of this study showed that the RVC and PFM movement varied with the position of the assessment. In contrast, MVC values are independent of the assessment position and can be selected according to clinical needs.

**Systematic review registration:**

PROSPERO, identifier CRD42022363734, https://www.crd.york.ac.uk/prospero/display_record.php?ID=CRD42022363734.

## Introduction

Urinary incontinence (UI), which is defined as an involuntary loss of urine (1), is the most common pelvic floor muscle (PFM) dysfunction in women (2, 3). In middle-aged and post-menopausal women, prevalence rates of urinary incontinence can reach 44–57% (4). Those who have UI may have physical and functional limitations, as well as a lower quality of life (5). The strength of the PFM is one of the triggers for urinary incontinence. PFM consists of the pelvic and urogenital diaphragm and plays a crucial role in the continence mechanism by engaging in rapid, strong, and reflexive contractions (6, 7). If the PFM is weak, the external urethral sphincter will lose support, leading to leakage. Strengthening the PFM is therefore essential for the prevention and treatment of urinary incontinence. The effectiveness of Kegel-based PFM training (PFMT) has been proven in clinical practice. It improves PFM strength (8) and accelerates the restoration of anatomical changes (including the position of the pelvis and the connective tissue morphology of the PFM) in postpartum women (9). PFMT is currently considered the first-line treatment for urinary incontinence (10), with a cure rate of 84% (11–13).

Selecting the appropriate body position for measurement will provide an accurate indication of the functional status of the PFM and facilitate subsequent PFMT-guided training. Some studies have shown that PFM functions vary in different body positions, such as standing, supine, and sitting position. There is evidence that resting voluntary contractions (RVC) values for PFM measured using the manometric were higher in the standing position than in the supine position, while maximum voluntary contraction (MVC) pressures, which represent the force-generating capacity of PFM (14), were significantly lower (15). In another trial, the measurements of MVC values yielded the opposite conclusion to the above study (16). The position that best reflects the functional status of the PFM in women with stress urinary incontinence remains controversial (17). Therefore, it is of practical importance to determine through meta-studies the interventional role of position in the assessment of the PFM (18) so that clinicians can make appropriate diagnoses and develop treatment plans.

Therefore, the research questions for this systematic review were:

1. Do body positions affect the measurement of PFM function?
2. If so, what are the factors and associated mechanisms by which body position affects measurement results?

## Materials and methods

The review was reported according to the Preferred Reporting Items for Systematic Reviews and Meta-Analyses (PRISMA) statement. Details of the protocol for this systematic review were pre-registered in the International Prospective Register of Systematic Reviews (PROSPERO): CRD42022363734.

### Search strategy

Two researchers independently searched for relevant articles. The full search strategy is presented in Appendix A1 in the supplementary material. A limited search was conducted in PubMed and EMBASE to identify and refine subject headings and keywords. Initially, a search strategy was designed using keywords, Medical Subject Heading (MeSH) terms, and free text words, such as Pelvic Floor, pelvic diaphragm, Posture, body position, upright, and so on. Additionally, keywords and subject headings were exhaustively combined using Boolean operators. Cross-sectional studies published between January 2000 and October 2023 that reported on the posture or PFM, or both were identified using EMBASE, PubMed, Scopus, web of science, and Cochrane. We also conducted a citation search of the included studies to further identify additional primary papers that might be eligible. Included cross-sectional studies had to involve the assessment of PFM function in at least two positions.

### Study selection

The included studies were required to have at least one of the MVC, RVC, and PFM movement outcomes. In addition, we included studies on females only. The search was limited to human studies. When multiple articles for a single study were present, we used the latest publication and supplemented it, if necessary, with data from the most complete or updated publication. Author disagreements were settled through consensus or following discussion with a third reviewer.

### Data extraction

Titles and abstracts of retrieved articles were screened for eligibility by two independent researchers. The full text was consulted if the abstract did not provide enough information for final evaluation. The following data were extracted from the original articles with a standardized data extraction form: author, published year, study location, characteristics of the participants (sample size, age, and duration of symptoms), outcomes, and variables that entered into the multivariable model as potential confounders such as measurement tools, measurement depth, rest time between positions, whether to receive verbal guidance in advance, etc. For each outcome, means and SDs were extracted. When necessary, means and SDs were calculated using available data (e.g., 95% CI or *p-*value) or information presented in the research. Author disagreements were resolved by consensus or after consultation with a third reviewer.

### Quality assessment

The quality of reporting for each study was performed by two researchers using the quality assessment tool for cross-sectional of The Agency for Healthcare Research and Quality (AHRQ) and any incongruity was discussed and resolved. This tool contains 9 questions. Each item on the AHRQ is answered as yes, no, or not reported, with only the answer “yes” scoring 1 and “no” and “not reported” scoring 0. 8–11 is considered high quality, and a score of 4–7 is considered moderate quality. A third author resolved any potential disagreement.

### Outcomes and data synthesis

The main outcomes were MVC, RVC, and PFM movement. MVC, which is the force exerted by the pelvic floor soft tissues to close the vaginal opening (19), can reflect the strength of the voluntary contraction of the PFM. PFM movement, defined as the movement of the bladder base during maximal voluntary contraction measured by transabdominal ultrasound (TAUS), serves as an indicator of PFM function (20–22). RVC force is the measure of PFM pressure at its original resting tone (14). All analyses were performed using Review Manager (RevMan, version 5.3 for Windows) to pool data for each outcome. Individual study effect sizes were expressed as standardized mean differences (SMDs), calculated as the difference in means between the two groups, divided by the pooled SD of the measurement. We calculated standardized mean differences (SMD) between subjects with different postures by meta-analysis with 95% CI to ascertain the potential effect of body position on outcomes (MVC, RVC, and PFM movement) in cross-sectional studies. In case of missing data, we tried to contact the corresponding author.

Due to the presence of confounding factors and large differences between the effect size indicators, heterogeneity may exist, so the random effects model is chosen. Heterogeneity between studies was assessed by I^2^, where I^2^ = 75–100% indicates may be considerable, I^2^ = 50–90% may be substantial, I^2^ = 30–60% may be moderate, and I^2^ < 40% may be low (23, 24). Moreover, to further investigate the sources of heterogeneity across the studies, subgroup analyses were performed according to cohorts (postpartum and asymptomatic women). Sensitivity analysis was conducted by excluding the studies that introduced significant heterogeneity to the analysis of each PFM function outcome, as long as 5 or more studies were available for inclusion.

## Results

### Compliance with the registered protocol

The registration scheme used MVC, RVC, and contraction holding period as outcome indicators. However, because of limitations in the number of included articles and considering the feasibility of meta-analysis, it was adjusted to MVC, RVC, and PFM movement in this article, and all effect sizes were treated equally. In addition to this, we conducted an update of the literature search in October 2023.

### Study selection

**Figure 1:**
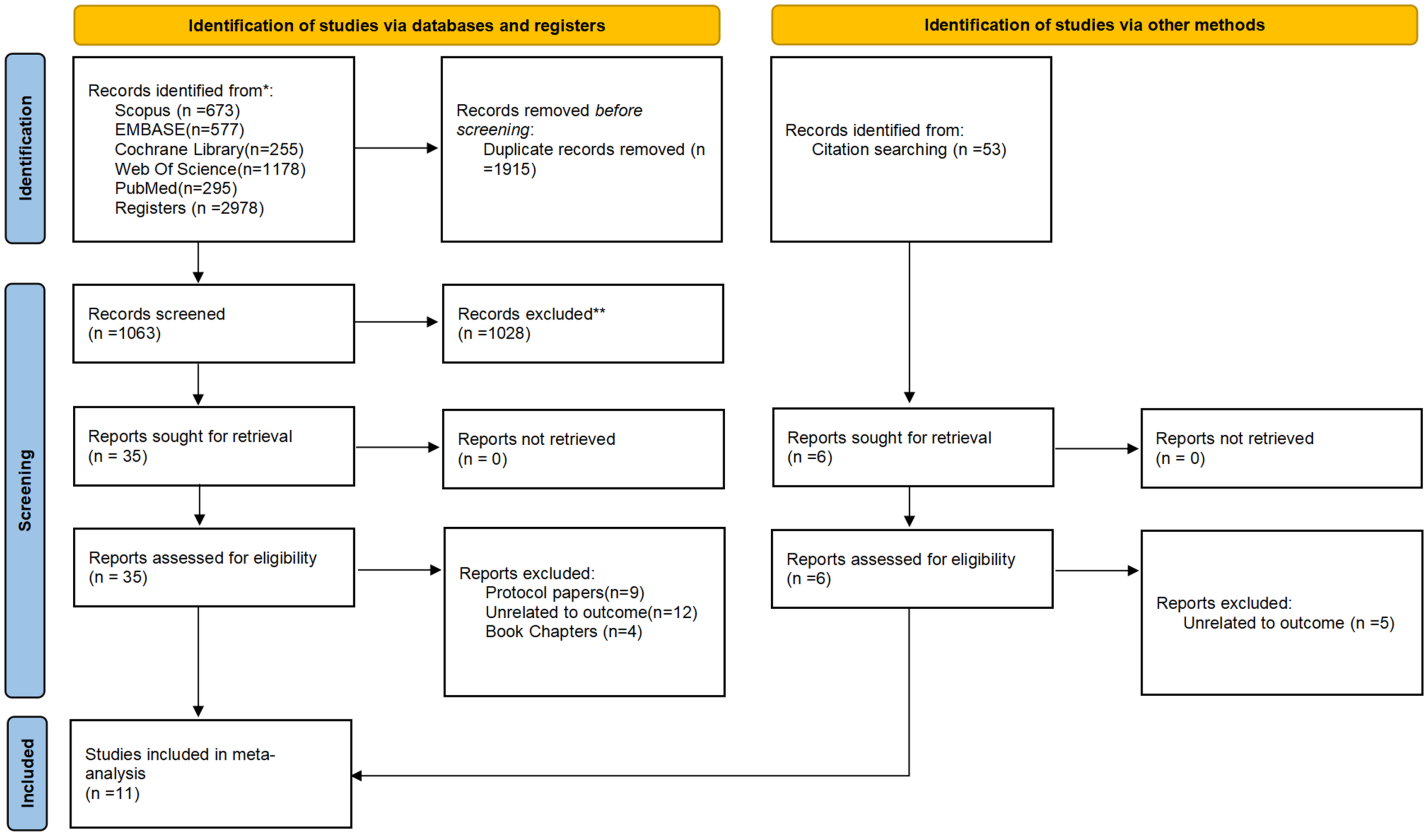
PRISMA flow diagram.

We identified a total of 2,978 articles, including five databases. After eliminating duplication and screening according to titles and abstracts, we selected 41 articles for further evaluation. In addition, relevant citation tracking was carried out to search and supplement. And after applying the inclusion and exclusion criteria, we retrieved 11 full-text articles for detailed review. The process of study selection is shown in Figure 1.

### Study characteristics

Study characteristics are provided in Table 1. The years of included research are from 2003 to 2022, of which three are from Australia (22, 25, 26), three are from Brazil (15, 27, 28), and the rest are from Norway (29), the United States (16), Switzerland (30), Iran (31), and Canada (32). In two of the trials (28, 31), outcomes of PFM function in asymptomatic and postpartum women were reported in groups. A total of 490 women were included with pelvic floor function status data, including 282 women with pelvic floor dysfunction and 208 healthy women. There is little difference in sample size (range: 15–89). All women are adult women (mean age 33.7 years [SD 11.3]). Six of the studies targeted women who had developed pelvic floor dysfunction after vaginal delivery, while seven included healthy adult women, who were considered to belong to healthy women because the cesarean section does not affect the PFM function of women.

**Table 1:** Characteristics of included studies.

Author (year), country	Participants	Age	Duration of symptoms	Measurement tools	Measured depth	Verbal guidance	PFM training	Number of measurements	Rest time between positions	Methodological quality AHRQ(11)
Bø K (2003), Norway	18, with symptoms of stress and mixed incontinence	31–64	Mean, 6.3 years	A fiberoptic microtip transducer connected to a balloon catheter	3.5 cm from the vaginal introitus	YES	YES	MVC (3, average); RVC (3, average)	NA	8/High
Daniel M (2005), USA	39 asymptomatic, continent women	Mean (SD), 45.8 (9.5)	NA	Instrumented vaginal speculum; a microtip catheter; 8F micro-tip dual sensor	7 cm similar to a standard speculum	YES	NA	MVC (3, average); RVC (3, average)	NA	9/High
Mary P (2006), Australia	20 women’s health physiotherapists	25–65	NA	The Peritron 9,300 perineometer; Acoustic Imaging Performa ultrasound unit	NA	YES	NA	MVC (3, the best); PFM movement (3, the best)	2 min	7/Moderate
Sally Mastwyk (2022), Australia	57 women with PFD	35–60	NA	The Blue Tran perineometer; a precursor prototype of the Peritron manometer	NA	NA	48, YES; 7, NO	MVC (3, the best); RVC (60s)	2 min	7/Moderate
Gameiro (2013), Brazil	50 healthy nulliparous volunteers	Mean, 23	NA	A perineometer with inflatable vaginal probe;	NA	YES	NA	MVC (3, average)	NA	9/High
Menta (2006), Brazil	73 had vaginal delivery and 22 had cesarean section.	20–32	NA	B-D brand, no. G93559, connected to a slightly tapered pear by a latex extension 80 cm long	NA	NA	NA	MVC (3, average)	15 s	7/Moderate
Helene (2019), Switzerland	17 young healthy nulliparous women	18–30	NA	Electromagnetic tracking system; transabdominal ultrasound	Midsagittal transabdominally touching the upper border of the pubic symphysis	YES	NA	MVC (3, average) PFM movement (3, average)	2 min	7/Moderate
Arab (2011), Iran	Continent (*n* = 15); incontinent (*n* = 15)	25–50	NA	Ultrasonix-ES500	NA	YES	NA	PFM movement (3, average)	10s	7/Moderate
Czyrnyj (2020), Canada	30 postpartum women	Mean (SD), 44.0 (12.8)	NA	An intravaginal dynamometer; ultrasound	Width along the lateral axis of the vagina, thickness along the anterior–posterior axis of the vagina, tail pointing up toward the pubis	YES	YES	MVC (3, average); RVC (3, 5 s)	90s	9/High
Kelly (2007), Australia	45 nulliparous female	Mean (SD), 23 (3)	NA	Transabdominal ultrasound	On the supra-pubic region in the transverse plane	NA	NA	PFM movement (3, average), Endurance (3, 60s)	60s	7/Moderate
Márcia (2022), Brazil	89 women with urinary incontinence	Mean (SD), 54 (11.0)	Mean, 3 years	NA	Above the level of the hymenal ring	YES	NO	MVC (3, the best)	5 min	9/High

NA, not available; MVC, maximal voluntary contractions; RVC, resting voluntary contractions; PFM, pelvic floor muscle; PFD, pelvic floor disorders.

### Risk of bias of included studies

The 11 observational cross-sectional studies included were assessed for risk of bias, and the results of AHRQ scale were shown in Supplementary Table S1 in the supplementary material. Only one trail is considered to be of high quality, and the rest are of medium quality. As all the included studies are cross-sectional studies at a certain time point, there is a lack of follow-up. And most studies do not explain how to deal with missing data, which leads to the risk of bias.

### Synthesis of results

#### The difference between standing and supine position

**Figure 2:**
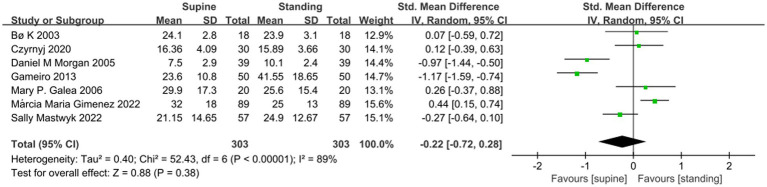
Comparison of MVC value results between supine and standing positions.

**Figure 3:**
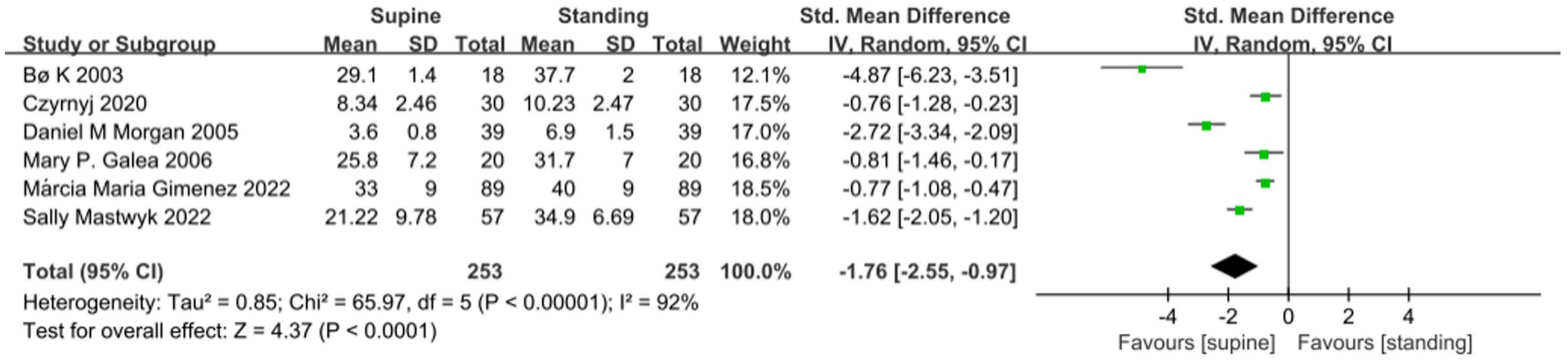
Comparison of RVC value results between supine and standing positions.

**Figure 4:**
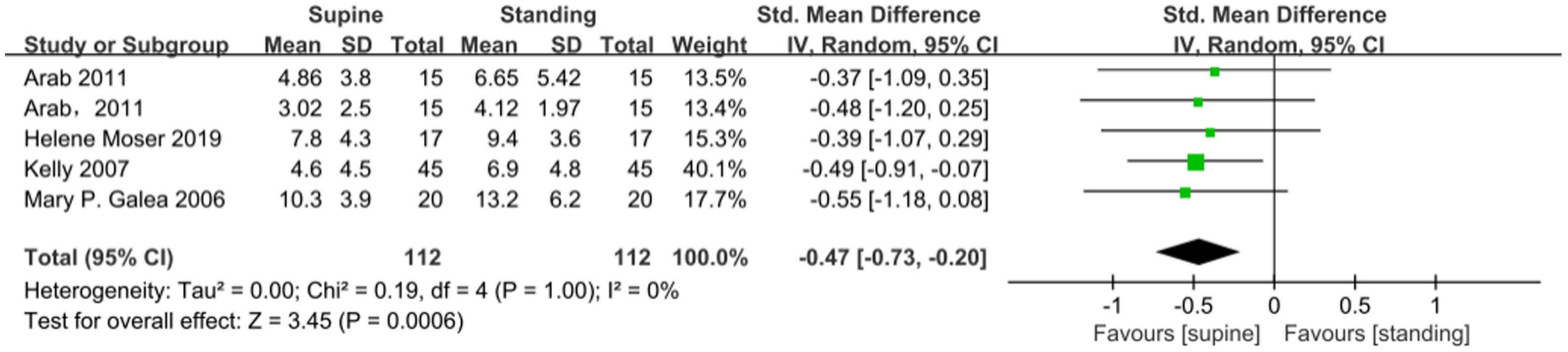
Comparison of PFM movement results between supine and standing positions.

A meta-analysis of seven studies showed no significant difference in the MVC of the PFM between the supine and standing position (SMD −0.22; 95% CI −0.72 to 0.28; *p* = 0.38; I^2^ = 89% Figure 2). A Meta-analysis of 6 studies showed that the RVC measured in standing position was significantly higher than that measured in supine position (SMD −1.76; 95% CI −2.55 to −0.97; *p* < 0.001; I^2^ = 92% Figure 3). A meta-analysis of five studies showed that PFM movement measured in standing position was significantly higher than that measured in supine position. (SMD-0.47; 95% CI −0.73 to 0.20; *P* = < 0.001; I^2^ = 0% Figure 4).

#### The difference between standing and sitting positions

**Figure 5:**
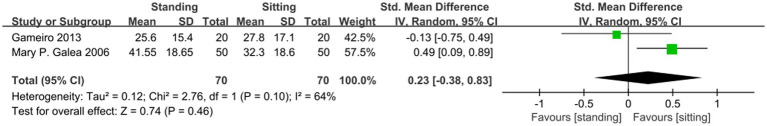
Comparison of MVC value results between standing and sitting positions.

A meta-analysis of two studies showed no significant difference in MVC values between standing and sitting positions (SMD 0.23; 95% CI −0.38 to 0.83; *p* = 0.46; I^2^ = 64% Figure 5). The included articles have not yet compared the differences in RVC between standing and sitting positions. Only one article compared the difference between PFM movement in the standing and sitting positions, and the results showed that the standing position was superior to the sitting position.

#### The difference between supine and sitting position

**Figure 6:**
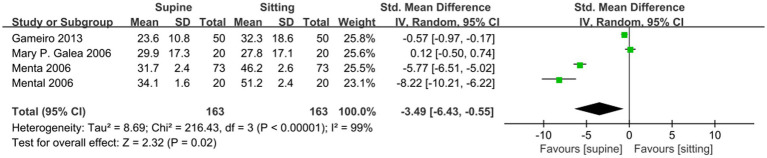
Comparison of MVC value results between supine and sitting positions.

**Figure 7:**
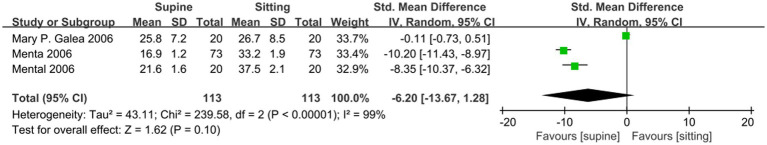
Comparison of RVC value results between supine and sitting positions.

A meta-analysis of four studies showed that MVC ability of PFM in sitting position was significantly better than that in supine position (SMD −3.49; 95% CI −6.43 to −0.55; *p* = 0.02; I^2^ = 99% Figure 6). A meta-analysis of three studies on the comparison of supine and sitting position assessment showed that there was no statistical difference between the two for the measurement of RVC (SMD −6.2; 95% CI −13.67 to 1.28; *p* = 0.1; I^2^ = 99% Figure 7). Only one article compared the difference in PFM movement between the supine and sitting positions, and the results showed that the sitting position was superior to the supine position.

#### Subgroup analysis and investigation of heterogeneity

Study heterogeneity could be further explained by women’s characteristics in the studies. Due to the limitation of the number of studies, only subgroup analyses of MVC and RVC in the supine and standing positions were conducted by the participant population. The existing research population is divided into two categories: postpartum and asymptomatic women (33). The subgroup analysis of MVC decreased the heterogeneity from considerable to substantial in postpartum population, but not the asymptomatic population (Supplementary Figure S1). Although the subgroup analysis of RVC has not decreased heterogeneity significantly (Supplementary Figure S2), the results vary in different populations. In postpartum population, the RVC value in standing position is better than that in supine position, but in asymptomatic population, there is no statistical difference between the two positions. These results suggest that different populations may be partly responsible for MVC heterogeneity, but whether populations are the cause of other outcomes heterogeneity is inconclusive. See Supplementary Figures S1, S2 in the supplementary material for a detailed forest plot.

Several factors explain the large heterogeneity of outcomes in this study. Firstly, there is a wide variation in the instruments used to measure between studies. Limited by age, some used a fiberoptic microtip transducer connected to a balloon catheter (29), while the Blue Tran perineometer (26) was used in recent studies. The anatomical position of these instruments is also variable (34), with some studies measuring 3.5 cm depending on the depth from the vaginal introitus (29), Some studies were set above the level of the hymenal ring (15) and even failed to find a clear description in some literature. Secondly, insufficient rest time can easily cause muscle fatigue in women, which affects the accuracy of the measurement results. However, the length of rest intervals between different body positions or measurements was not uniform among the included studies.

## Discussion

The current systematic review showed that the RVC and PFM movement values measured in the standing position were higher compared to the supine position, while the MVC values were not significantly different between the two. Few articles were published comparing the standing position with the seated position, and only two articles were comparing MVC values, which showed no statistically significant results. Only one study compared the differences in PFM movement measurements and the results showed that the standing position was superior to the sitting position. The results in the sitting position showed that both MVC and PFM movement values were better than in the supine position, while RVC was not statistically different.

RVC of the PFM in the standing position was significantly higher than that in the supine position, indicating that the resting pressure of the PFM in the supine position did not accurately reflect the muscle state. The data obtained from the evaluation in the upright posture captured the natural relaxation position and movement of PFM in daily life, reflecting the functional state of the lower PFM most of the time (26). In normal healthy people, contraction of the PFM before exertion is a natural response and does not require conscious exertion, which is closely related to the functional significance represented by the RVC value. Therefore, standing posture may be more useful when investigating the automatic function of PFM in response to increased intra-abdominal pressure and muscle fatigue (35, 36). Compared with the supine position, standing position is also more helpful to analyze the PFM function and its related mechanisms in women with stress urinary incontinence (29). The results of the study showed that displacement of pelvic floor elevation differed when measured in the standing and supine positions. PFM movement was significantly higher in the former. It may be due to the improvement of women’s proprioception by standing measurement, or by factors such as gravity (37) and intra-abdominal pressure (38, 39). However, there is no precise machine to evaluate intra-abdominal pressure, so it is impossible to confirm and exclude the specific effect of intra-abdominal pressure on the evaluation of pelvic floor function. On the other hand, a major drawback of PFM movement is that there is no fixed anatomical landmark, and its starting point is dynamically changing (16). Therefore, the resting tension of the muscle may be a potential confounding factor in the measurement of elevation displacement (17).

Our study is the first meta-analysis of the correlation between body positions and PFM contractile function. The evaluation and comparison of functional PFM in different postures can bring new understanding to the evaluation of pelvic floor function. A precise description of the assessment position should aid the standardization of measurement and enable comparisons of findings between studies. For the MVC ability of female PFM, there is no statistical significance in a variety of positions, indicating that it can be performed in a position that is convenient for therapists and comfortable for women according to clinical needs. Moreover, studying how PFM activity changes with postural changes will help to better understand the impact of body position on women’s symptoms and thus find the best PFM training program to best restore functional impairment (17).

Our study has several limitations. All effect sizes are treated equally in our analyses, regardless of whether these outcomes were primary or secondary in the original studies, which may have influenced our findings because some studies may have been underpowered to detect a significant effect on some outcomes. Secondly, these findings are significantly affected by the nature of the cross-sectional studies, which are highly susceptible to biases. The number of participants in the systematic review and especially meta-analysis was relatively small. Various types of bias may have influenced the study findings. For example, there are few studies included in this Meta-analysis, the experimental sample size is small, and the period of each trial is large. On the other hand, the body positions involved in each study are limited, which can also be classified in detail according to pelvic position, such as whether the pelvis is accompanied by a backward tilt in the sitting position, and so on. In addition, the included studies were clinically heterogeneous in their choice of clinical methods, such as the instruments used, and the degree of attention paid to the correct contraction of PFM. Although the results show greater RVC measurements in the standing position, the standing position has the following disadvantages. First of all, due to the visual field, the therapist may need to spend more time and the operation is more troublesome (33). Secondly, it is easier to cause the symptoms of women with pelvic floor dysfunction and cause embarrassment and discomfort of women.

More high-quality studies are needed, given the small number of trials included in our review and the high heterogeneity. The PFM could be measured separately before and after a period of a training intervention to ensure that the women can contract correctly before comparing the effect of different positions on the measurement results. In addition, the future classification of positions could be enriched with categories of positions such as sitting with pelvic tilt or not. An expert consensus could be developed in the future to standardize the use of instruments and reduce errors in measurement results. Future studies ought to verify the potential impact of instrument placement and intra-abdominal pressure on measurement results (40–42). In addition, the subjective feelings of women during training or evaluation can be considered in the following research (43).

## Conclusion

The results of this study showed that RVC, and PFM movement measurements, which reflect female pelvic floor function, varied with the assessment position, confirming the importance of the position used when recording the assessment. However, the MVC value is not related to the evaluation position and can be selected according to clinical needs.

## Data availability statement

The original contributions presented in the study are included in the article/Supplementary material, further inquiries can be directed to the corresponding author/s.

## Author contributions

LH: conceptualization, methodology, data curation, software, writing-original draft preparation. Z-YZ: conceptualization, methodology, data curation. HL: methodology, software. MG: visualization, investigation. X-QW: methodology, software. X-QD: writing- reviewing, supervision. Z-LL: writing- reviewing, supervision, editing. All authors contributed to the article and approved the submitted version.
