# Supplementary material for: Most of the pelvic floor muscle functions in women differ in different body positions, yet others remain similar: systematic review with meta-analysis
Source: Front Med (Lausanne). 2023 Nov 6;10:1252779. doi: 10.3389/fmed.2023.1252779 (PMC10662015; doi:10.3389/fmed.2023.1252779)
Supplement: Supplementary file 1 [file Table_1.DOCX]

| **Study** | D1 | D2 | D3 | D4 | D5 | D6 | D7 | D8 | D9 | D10 | D11 | **Score** |
| --- | --- | --- | --- | --- | --- | --- | --- | --- | --- | --- | --- | --- |
| **Bø K, (2003)^26^, Norway** | * | * | * | * | × | * | × | * | × | × | × | 6/Moderate |
| **Daniel M^27^ (2005), The United States of America** | * | * | * | * | × | * | * | * | × | × | × | 7/Moderate |
| **Mary P^30^ (2006), Australia** | * | * | * | * | × | * | * | * | × | × | × | 7/Moderate |
| **Sally Mastwyk^23^ (2022), Australia** | * | * | * | * | × | * | * | * | × | × | × | 7/Moderate |
| **Gameiro^19^ (2013), Brazil** | * | * | * | * | × | * | × | * | × | * | × | 7/Moderate |
| **Menta,^24^ (2006), Brazil** | * | * | * | * | × | * | × | × | × | × | × | 5/Moderate |
| **Helene^28^ (2019), Switzerland** | * | * | * | * | × | * | × | * | × | × | × | 6/Moderate |
| **Arab^13^ (2011), Iran** | * | * | * | * | × | * | × | * | × | × | × | 6/Moderate |
| **Czyrnyj^29^ (2020), Canada** | * | * | * | * | × | * | × | × | × | × | × | 5/Moderate |
| **Kelly^21^ (2007), Australia** | * | * | * | * | × | * | * | * | × | × | × | 7/Moderate |
| **Márcia^25^ (2022), Brazil** | * | * | * | * | × | * | * | * | × | * | × | 8/High |

**Supplementary Table1. Methodological Quality Assessed on AHRQ.**

D1. Define the source of information.

D2. List inclusion and exclusion criteria for exposed and unexposed subjects (cases and controls) or refer to previous publications.

D3. Indicate time period used for identifying women.

D4. Indicate whether or not subjects were consecutive if not population-based.

D5. Indicate if evaluators of subjective components of study were masked to other aspects of the status of the participants.

D6. Describe any assessments undertaken for quality assurance purposes.

D7. Explain any women exclusions from analysis.

D8. Describe how confounding was assessed and/or controlled.

D9. If applicable, explain how missing data were handled in the analysis.

D10. Summarize women response rates and completeness of data collection.

D11. Clarify what follow-up, if any, was expected and the percentage of women for which incomplete data or follow-up was obtained.
