# Supplementary material for: Most of the pelvic floor muscle functions in women differ in different body positions, yet others remain similar: systematic review with meta-analysis
Source: Front Med (Lausanne). 2023 Nov 6;10:1252779. doi: 10.3389/fmed.2023.1252779 (PMC10662015; doi:10.3389/fmed.2023.1252779)
Supplement: Supplementary file 2 [file Data_Sheet_1.docx]

Appendix A1. Example search strategy.

Databases: EMBASE, PubMed, Scopus, web of science, and Cochrane

The search strategy for PubMed was shown below. It was adapted for the other databases.

(("Pelvic Floor"[MeSH Terms] OR "Pelvic Floor"[Title/Abstract] OR "floor pelvic"[Title/Abstract] OR "pelvic diaphragm"[Title/Abstract] OR "diaphragm pelvic"[Title/Abstract] OR (("Diaphragm"[MeSH Terms] OR "Diaphragm"[All Fields] OR "contraceptive devices, female"[MeSH Terms] OR ("contraceptive"[All Fields] AND "devices"[All Fields] AND "female"[All Fields]) OR "female contraceptive devices"[All Fields] OR "Diaphragms"[All Fields] OR "diaphragm s"[All Fields] OR "diaphragmal"[All Fields]) AND "Pelvic"[Title/Abstract]) OR "pelvic diaphragms"[Title/Abstract]) AND ("Prone"[Title/Abstract] OR "patient positioning"[Title/Abstract] OR "body position"[Title/Abstract] OR "upright"[Title/Abstract] OR ("Posture"[MeSH Terms] OR "Posture"[Title/Abstract] OR "postures"[Title/Abstract] OR "lying"[Title/Abstract] OR "lay"[Title/Abstract] OR "stand"[Title/Abstract] OR "sit"[Title/Abstract]))) AND (2000:2022[pdat])
