# Supplementary figures and images for: Most of the pelvic floor muscle functions in women differ in different body positions, yet others remain similar: systematic review with meta-analysis
Source: Front Med (Lausanne). 2023 Nov 6;10:1252779. doi: 10.3389/fmed.2023.1252779 (PMC10662015; doi:10.3389/fmed.2023.1252779)

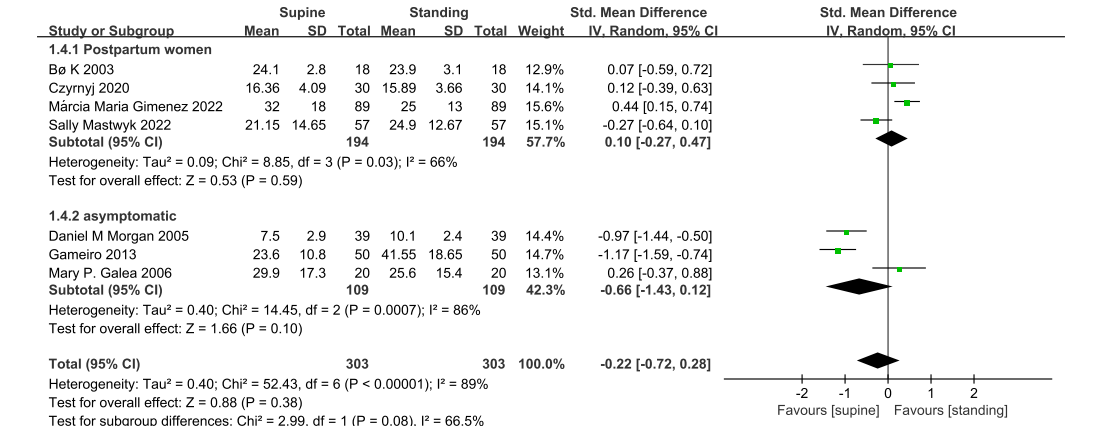

Supplement: Supplementary file 3 [file Image_1.TIF]

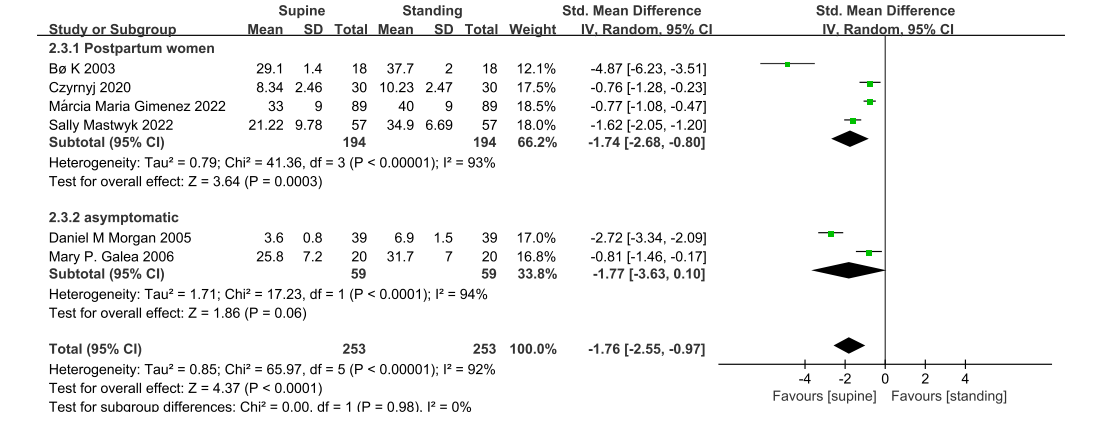

Supplement: Supplementary file 4 [file Image_2.TIF]
